# A Predictive Model for Knee Joint Replacement in Older Women

**DOI:** 10.1371/journal.pone.0083665

**Published:** 2013-12-11

**Authors:** Joshua R. Lewis, Satvinder S. Dhaliwal, Kun Zhu, Richard L. Prince

**Affiliations:** 1 School of Medicine and Pharmacology, University of Western Australia, Perth, Western Australia, Australia; 2 Department of Endocrinology and Diabetes, Sir Charles Gairdner Hospital, Perth, Western Australia, Australia; 3 School of Public Health, Curtin University, Perth, Western Australia, Australia; Universidad Europea de Madrid, Spain

## Abstract

Knee replacement (KR) is expensive and invasive. To date no predictive algorithms have been developed to identify individuals at high risk of surgery. This study assessed whether patient self-reported risk factors predict 10-year KR in a population-based study of 1,462 women aged over 70 years recruited for the Calcium Intake Fracture Outcome Study (CAIFOS). Complete hospital records of prevalent (1980-1998) and incident (1998-2008) total knee replacement were available via the Western Australian Data Linkage System. Potential risk factors were assessed for predicative ability using a modeling approach based on a pre-planned selection of risk factors prior to model evaluation. There were 129 (8.8%) participants that underwent KR over the 10 year period. Baseline factors including; body mass index, knee pain, previous knee replacement and analgesia use for joint pain were all associated with increased risk, (P < 0.001). These factors in addition to age demonstrated good discrimination with a C-statistic of 0.79 ± 0.02 as well as calibration determined by the Hosmer-Lemeshow Goodness-of-Fit test. For clinical recommendations, three categories of risk for 10-year knee replacement were selected; low < 5%; moderate 5 to < 10% and high ≥ 10% predicted risk. The actual risk of knee replacement was; low 16 / 741 (2.2%); moderate 32 / 330 (9.7%) and high 81 / 391 (20.7%), P < 0.001. Internal validation of this 5-variable model on 6-year knee replacements yielded a similar C-statistic of 0.81 ± 0.02, comparable to the WOMAC weighted score; C-statistic 0.75 ± 0.03, P = 0.064. In conclusion 5 easily obtained patient self-reported risk factors predict 10-year KR risk well in this population. This algorithm should be considered as the basis for a patient-based risk calculator to assist in the development of treatment regimens to reduce the necessity for surgery in high risk groups such as the elderly.

## Introduction

Osteoarthritis results in chronic pain, deformity, disability and loss of quality of life with one third of all people above the age of 85 suffering from this debilitating condition [1]. Despite being a cost-effective treatment for osteoarthritis [2] and having relatively low revision rates [3], knee replacements (KR) are a major burden on healthcare systems worldwide [1,4]. 

Another major burden to the developed world’s healthcare system is obesity [5] with the prevalence increasing by more than 2.5 times over the past 20 years [6]. Lifestyle changes to diet and physical activity have contributed to this rapid increase of obese people with abdominal obesity increasing the risk of serious diseases such as type II diabetes, metabolic syndrome and cardiovascular disease [7]. Obesity is also strongly associated with knee osteoarthritis and lower limb pain [8,9,10] however there are conflicting findings as to whether obesity is associated with knee joint replacement [11,12,13,14]. 

Few population-based longitudinal studies have investigated the predictive value of self-reported and anthropometric measures on KR and to date no predictive risk models for KR exist. The development of a standardized predictive model for knee replacement will allow testing of novel risk factors and to assist in the development of treatment regimens to reduce the necessity for this expensive and invasive surgery. We therefore used 10-year procedure codes and hospital discharge data from the Western Australia Data Linkage System (WADLS) in conjunction with baseline data collected in the CAIFOS study to identify easily obtained patient self-reported data that predicts 10-year knee joint replacement in a community based setting. 

## Methods

The primary outcome was a knee replacement (KR) procedure with pre-planned easily obtained self-reported variables including baseline age, knee pain, physical activity, calcium intervention group, prevalent knee replacement and analgesia use for joint pain. Anthropometric variables were then included individually into the multivariable receiver operator curve (ROC) analysis to assess whether their inclusion significantly improved predictive models.

### Study participants

 The participants were recruited in 1998 to a 5-year prospective, randomized, controlled trial of oral calcium supplements to prevent osteoporotic fracture [15]. Women were recruited from the Western Australian general population of women aged over 70 years by mail using the electoral roll. Over 99% of Australians of this age are registered on the roll. Of the 5,586 women who responded to a letter inviting participation 1,510 women were willing and eligible and of these 1,500 women were recruited for the study. Participants were ambulant and did not have any medical conditions likely to influence 5-year survival. They were excluded if they were receiving bone-active agent, including hormone replacement therapy. Participants were similar in terms of baseline disease burden and medications compared to the whole population of this age but they were more likely to be from higher socio-economic groups [15]. In the 5 years of the trial, participants received 1.2 g of elemental calcium as calcium carbonate daily or a matched placebo. Participants were subsequently included in a 5-year follow-up study of ageing. Complete clinical and anthropometrical data were available in 1,478 participants of these 16 participants had total knee replacements performed on both knees and were excluded leaving 1,462 participants. The human ethics committee of the University of Western Australia approved the study and written informed consent was obtained from all participants prior to recruitment.

### Baseline measurements

At baseline, information was obtained from the patient on their previous medical history and current medications, the participants were asked to verify this information with their General Practitioner where available. This data was then coded using the International Classification of Primary Care – Plus (ICPC-Plus) method [16]. The coding methodology allows aggregation of different terms for similar pathologic entities as defined by the ICD-10 coding system. Analgesic medications used to treat joint pain at baseline included non-steroidal anti-inflammatory drugs (NSAID’s) and paracetamol. Physical activity level was assessed by questionnaire [17,18], and calculated in kcal/day using a validated method utilising body weight, questions on the number of hours and type of physical activity and energy costs of such activities [19,20]. Because of the randomisation to calcium or placebo for the first five years of the study a variable named “calcium intervention group” capturing this randomisation was included in the model. Information on knee joint pain frequency and site was collected by a questionnaire at baseline. In the questionnaire, subjects were asked to select one of five categories that best describes the frequency of the pain they experienced at the knee over the previous year: (1) never, (2) less than once a month, (3) once a month to once a week, (4) once a week to once a day, and (5) once a day or more. These categories were then transformed into three categories; frequency < once a month (infrequent); ≥ one a month to < once a day (frequent) or ≥ once a day (daily).At baseline weight was assessed using digital scales with participants wearing light clothes and no shoes, height was assessed using a stadiometer and the body mass index (BMI) was calculated in kg/m^2^. 

### Previous knee replacement

Knee replacement procedures in patients with a primary diagnosis of osteoarthritis were retrieved from the Western Australian Data Linkage System (WADLS) hospitalisation records for each of the study participants from 1980 to 1998, when participants were entered into the CAIFOS study. WADLS provides a complete validated record of every participant’s primary diagnosis and up to 21 additional diagnoses of hospitalizations and procedure codes within Western Australia. Events were defined using primary diagnosis and procedure codes from the International Classification of Diseases, Injuries and Causes of Death Clinical Modification (ICD-9-CM procedure code 81.54) [21]. 

### Incident knee replacement

The primary outcome was KR procedures excluding those with revisions of previous surgery and those with a primary diagnosis other than osteoarthritis of the knee. Incident knee replacement procedures were retrieved from the Western Australian Data Linkage System (WADLS) hospitalization record for each of the study participants from 1998, until 10 years after their baseline visit. Events were defined using primary diagnosis and procedure codes from the International Classification of Diseases, Injuries and Causes of Death Clinical Modification (ICD-9-CM) [21] and the International Statistical Classification of Diseases and Related Health Problems, 10^th^ Revision, Australian Modification (ICD-10- AM) [22]. The procedure codes were classified according to the Australian Institute of Health and Welfare intervention codes (ICD-9-CM procedure code 81.54 and ICD-10-AM procedure codes) for primary partial (49517-00) and total knee replacements (49518-00, 49519-00, 49521-00, 49521-01, 49521-02, 49521-03, 49524-00, 49524-01, 49534-00), [1]. 

### Internal validation

The model was re-tested at 48 months for a shortened duration of 6 years (2002-2008) in the 1,119 participants who attended the 2002 clinic visit and compared to WOMAC for internal validation. Information on knee joint pain frequency was collected by questionnaire at 60 months.

### Western Ontario and Mcmaster University Osteoarthritis Index (WOMAC)

The Western Ontario and Mcmaster University Osteoarthritis Index (WOMAC) was completed at the 48 month clinic visit. This scoring system assesses pain (0 to 20), stiffness (0 to 8) and physical function (0 to 68), with higher scores indicating more debilitating osteoarthritis [23]. 

### Statistical analysis

The primary outcome was a KR procedure with a selection of pre-planned patient self-reported data variables including age, knee pain at baseline, physical activity, calcium intervention group, prevalent knee replacement and analgesia use for joint pain at baseline. Physical activity, height and calcium intervention were non-significant in the model and were excluded from further analyses. As age > 70 years was a selection variable in the CAIFOS study it was retained despite being non-significant in the model to provide age-adjusted effects of predictor variables. Results are presented as either an Odds Ratio (OR) or Hazard Ratio (HR) and associated 95% confidence intervals. P values less than 0.05 in two tailed tests were considered statistically significant. The data was analysed using PASW software (version 18, SPSS Inc., Chicago, IL), STATA (version 11 StataCorp LP, College Station, TX) and SAS (Version 9, SAS Institute Inc., Chicago, IL).

## Results

Over the 10 years of the study there were 129 (8.8%) individuals who had KR with a primary diagnosis of osteoarthritis. Of these there were 119 total knee replacements and 10 partial knee replacements. The effects of the selected baseline characteristics of the participants by knee replacement status are shown are shown in Table 1. In those subsequently requiring KR there was a higher baseline prevalence of knee pain, consumption of analgesia for joint pain, previous knee replacement and higher body mass index. Age, height, physical activity and calcium intervention group were not significantly different between those with and without knee replacement. 

**Table 1:** Baseline characteristics.

**Characteristics**	**No knee replacement (n = 1,333)**	**10-year knee replacement (n = 129)**	**P value**
**Age (years)**	75.2 ± 2.7	75.1 ± 2.5	0.695
**Physical Activity (Kcal)**	141 ± 153	138 ± 169	0.806
**Knee pain**			
**Infrequent**	**901 (67.6)**	**35 (27.1)**	**<0.001**
**Frequent**	**259 (19.4)**	**39 (30.2)**	
**Daily**	**173 (13.0)**	**55 (42.6)**	
**Analgesia use for joint pain (yes)**	**402 (30.2)**	**76 (58.9)**	**<0.001**
**Previous knee replacement (yes)**	**31 (2.3)**	**13 (10.1)**	**<0.001**
**Calcium intervention (yes)**	687 (51.5)	60 (46.5)	0.473
**Anthropometric measures**			
**Weight (kg)**	**67.9 ± 12.3**	**74.9 ± 13.0**	**<0.001**
**Height (cm)**	158.8 ± 6.0	159.2 ± 6.0	0.385
**Body Mass Index (kg/m^2^)**	**26.9 ± 4.7**	**29.5 ± 4.8**	**<0.001**

All values are mean ± standard deviation for continuous variables or number and percentage for categorical variables.

The selected variables were then entered into the model as continuous variables, with the exception of prevalent knee replacement and analgesia use for joint pain, which were entered as dichotomous (yes / no) variables and knee pain at baseline which was entered as three groups (infrequent / frequent / daily). Calcium intervention and physical activity were not significant in the model and were excluded from further analyses (Table 2). Similarly including the 41 participants with clinical diagnosis of osteoarthritis of the knee at baseline or socioeconomic status did not significantly improve the model (improvement to the C-statistic + 0.007, P = 0.162 and + 0.001, P = 0.758 respectively). 

**Table 2:** Unstandardized regression coefficients and odds ratio of individual variables tested for 10-year total knee replacement prediction.

**Clinical measures (n = 1,462)**	**β**	**X^2^**	OR* (95% CI)	**P value**
**Age (years)**	-0.013	0.154	0.99 (0.92-1.06)	0.695
**Prevalent knee replacement (yes)**	**1.549**	**20.237**	**4.71 (2.40-9.22)**	**<0.001**
**Analgesia use for joint pain (yes)**	**1.200**	**40.480**	**3.32 (2.29-4.81)**	**<0.001**
**Knee pain (infrequent/frequent/daily**)	**1.043**	**86.817**	**2.84 (2.28-3.53)**	**<0.001**
**Calcium intervention (yes)**	-0.151	0.783	0.86 (0.62-1.20)	0.376
**Height (cm)**	0.013	0.757	1.01 (0.98-1.05)	0.384
**Weight (kg)**	**0.040**	**35.128**	**1.04 (1.03-1.05)**	**<0.001**
**Body mass index (kg/m^2^)**	**0.100**	**33.071**	**1.11 (1.07-1.14)**	**<0.001**

^*^ Odds Ratio (OR) with “no” category as the referent.

### Body mass index

**Figure 1:**
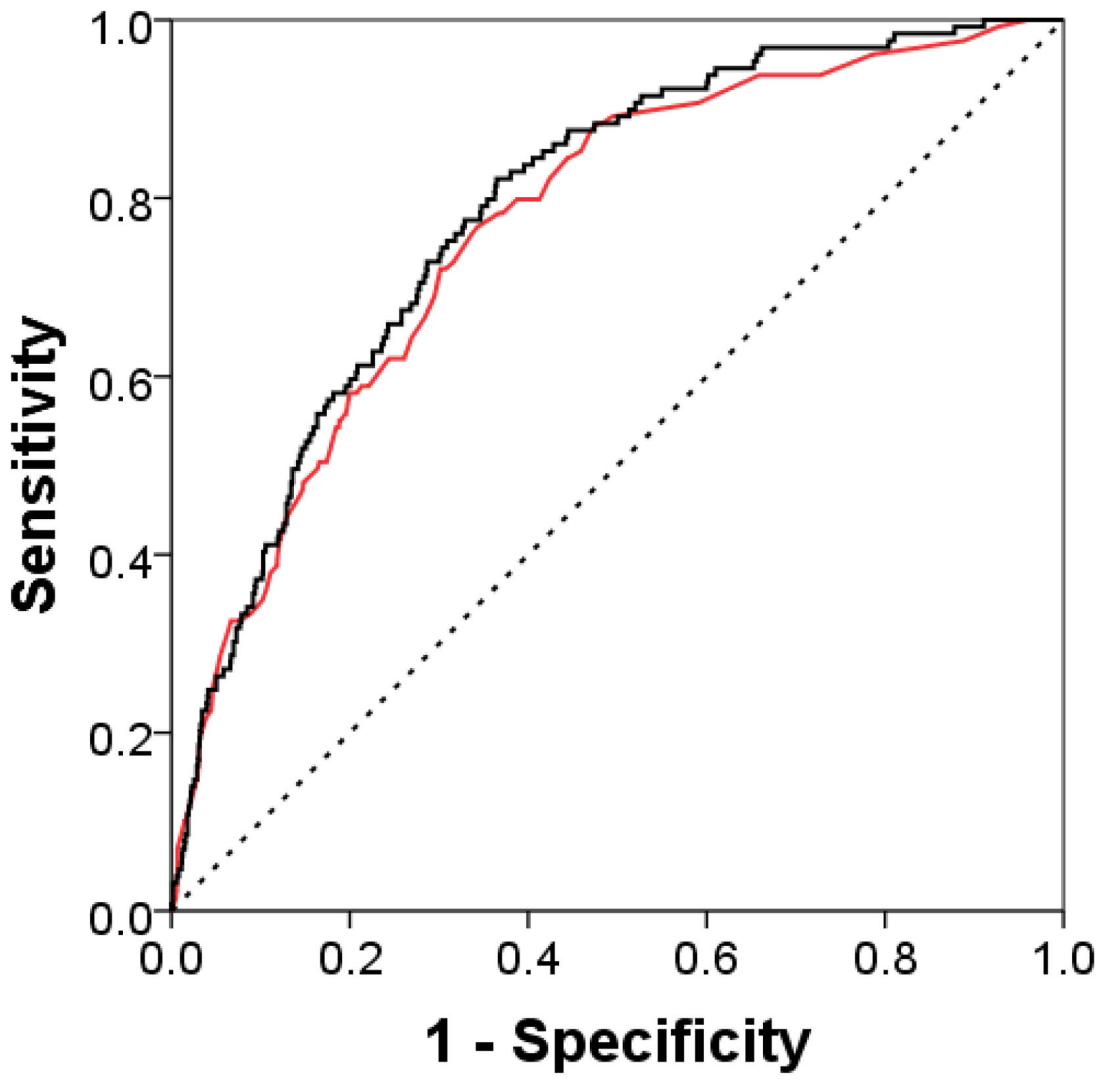
Receiver operator characteristic (ROC) curve for the 5-variable predictive model. The model includes age, knee joint pain at baseline, analgesia use for joint pain at baseline, prevalent knee joint replacement and body mass index; C-statistic 0.787 ± 0.019 (black line) or without body mass index C-statistic 0.768 ± 0.021 (red line).

The model was significantly improved with the addition of body mass index (improvement to the C-statistic + 0.019, P = 0.011, Figure 1).The beta coefficients for the patient self-reported variables in the final model are shown in Table 3.

**Table 3:** Unstandardized regression coefficients and odds ratio of the variables in the final 5-variable model for 10-year total knee replacement prediction.

**Clinical measures (n = 1,462)**	**β**	**X^2^**	OR* (95% CI)	**P value**
**Age (years)**	-0.002	0.004	1.00 (0.93-1.07)	0.949
**Prevalent knee replacement (yes)**	**0.731**	**3.936**	**2.08 (1.01-4.28)**	**0.047**
**Analgesia use for joint pain (yes)**	**0.682**	**11.208**	**1.98 (1.33-2.95)**	**0.001**
**Knee pain (infrequent/frequent/daily**)	**0.854**	**51.260**	**2.35 (1.86-2.97)**	**<0.001**
**Body mass index (kg/m^2^)**	**0.069**	**12.94**	**1.07 (1.03-1.11)**	**<0.001**

^*^ Odds Ratio (OR) with “no” category as the referent.

### Sensitivity analysis

The model was further examined in individuals without previous knee replacement, without daily knee pain and without analgesia for joint pain at baseline. In the 1,418 participants without prevalent knee replacement there was good discrimination with a C-statistic of 0.780 ± 0.020, similarly in those without daily knee joint pain (n = 1,234) there was good discrimination with a C-statistic of 0.762 ± 0.027 and in the 984 participants without analgesia use for joint pain at baseline the C-statistic was 0.788 ± 0.031. 

### Calibration

**Figure 2:**
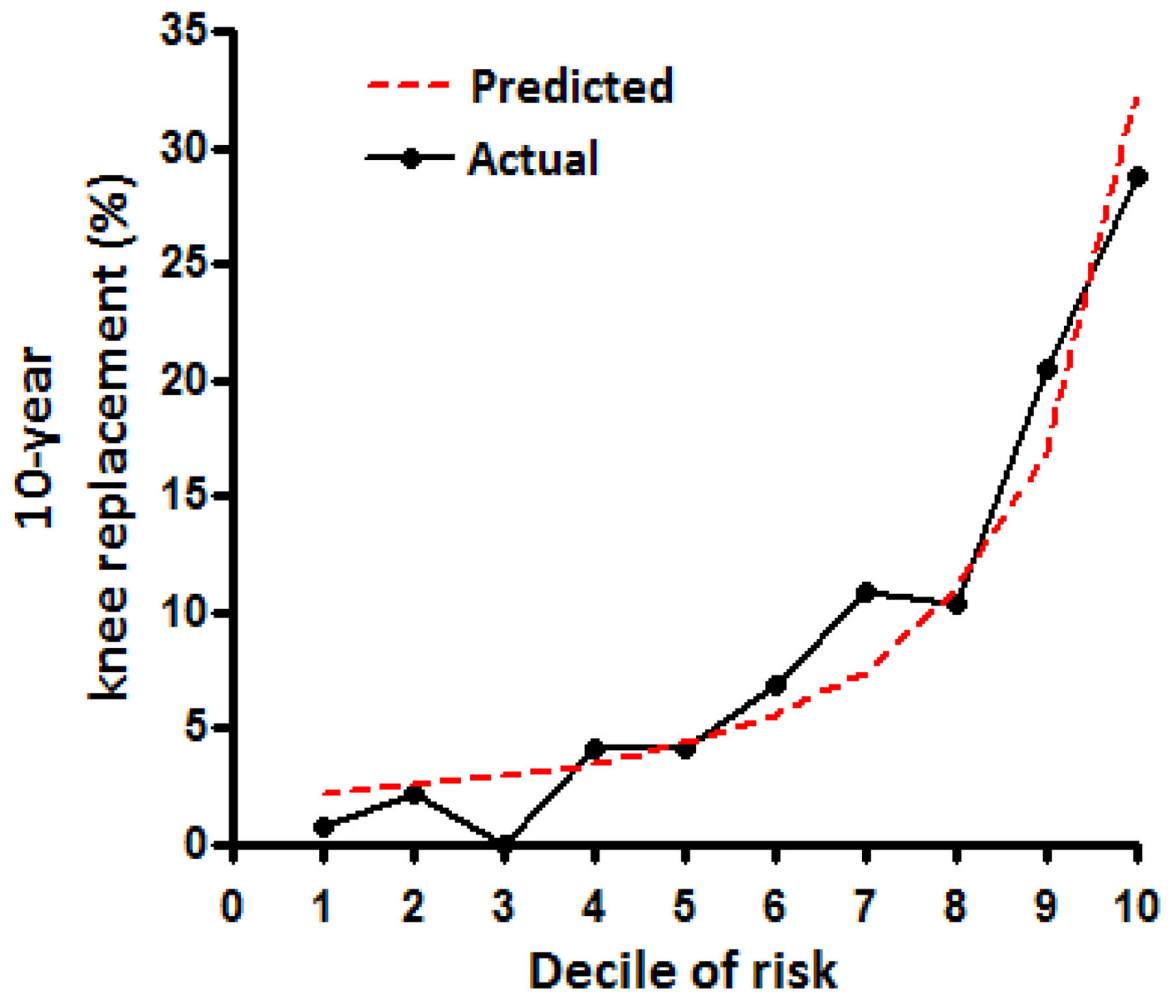
Predicted 10-year risk vs. **actual 10-year knee replacement**. Categorized by deciles of predicted risk (n = 1,462). Model calibration tested by Hosmer-Lemeshow Goodness-of-Fit test, P = 0.179.

The calibration of the final model was tested by separating participants into deciles of predicted 10-year knee replacement risk compared to the actual risk (Figure 2). This analysis demonstrated good calibration with the actual risk of KR in these categories, as demonstrated by the non-significant Hosmer-Lemeshow test. For clinical recommendations, three categories of risk for KR were selected, low (

**Figure 3:**
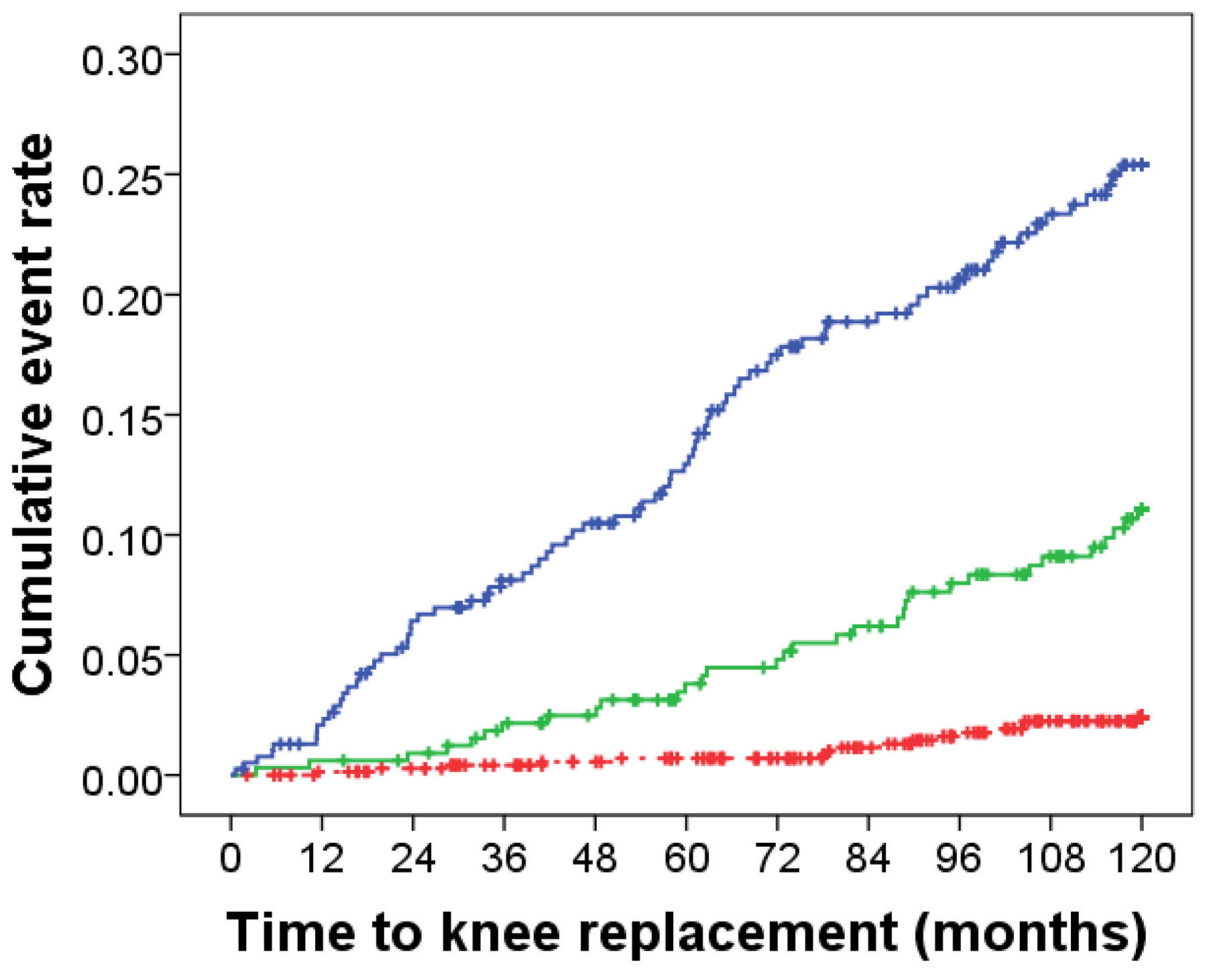
Kaplan Meier hazard plot for 10-year knee joint replacement. Categorized into 3 categories of predicted risk of 10-year knee replacement; red dotted line; low risk (< 5%); blue line moderate risk (5 - < 10%) and black line, high risk (≥ 10%), P < 0.001 when compared by the log-rank test.

< 5% over 10 years); moderate (5 to < 10% over 10 years) and high (≥ 10% over 10 years). The actual risk of knee replacement in the three groups was; low 16 / 741 (2.2%); moderate 32 / 330 (9.7%) and high 81 / 391 (20.7%), see Figure 3

. 

### Internal validation

The 5-variable model was then re-tested at 48 months in the 1,119 participants who attended the 48 and 60 month clinic visit and filled in the questionnaires. There were 65 participants that underwent KR in the subsequent 6 years. Discrimination and calibration of the 5-variable model was then tested and compared to the weighted WOMAC score. The model again demonstrated good discrimination with a C statistic of 0.810 ± 0.023 and calibration demonstrated by the non-significant Hosmer-Lemeshow goodness of fit test, P = 0.185. The C statistic for the weighted WOMAC score was 0.748 ± 0.030, (mean difference -0.062, P = 0.064), see Figure S1 and Table S1. 

## Discussion

In this prospective population-based study of elderly women we found that 5 simply evaluated variables including joint pain, age, body mass index, previous knee replacement and analgesia use for joint pain provided good clinical utility in predicting future 10-year KR. This model also demonstrated good discrimination with a C-statistic of 0.79 for 10-year KR. This C-statistic is comparable to other 10-year risk prediction models for major osteoporotic fracture (0.68) and hip fracture (0.75) and coronary heart disease prediction in men (0.74) and women (0.77) [24,25,26]. The 5-variable model was then internally validated using the 48 month risk factors and compared to the WOMAC score and found to be comparable to the more complicated WOMAC test [23]. We developed this community based risk calculator for the prediction of KR in the elderly using non-radiographic self-reported variables primarily as a “basic” model to test novel metabolic, biochemical and hormonal risk factors as there is currently no prognostic model available. Secondly we developed this model to allow clinical researchers to identify high risk individuals from large population based cohorts for randomized controlled trials of novel treatments and early intervention to prevent progression to KR. 

Interestingly in our cohort when clinical diagnosis of osteoarthritis of the knee at baseline was added to the model it did not significantly increase the discrimination perhaps due to the small number of clinically diagnosed knee OA cases. Similarly socioeconomic status was not associated with the decision to undergo surgery which perhaps relates to Australia’s national healthcare system that provides for the joint replacement procedures irrespective of the ability of the patient to pay for the surgery, similar to that of the US Medicare system for people over the age of 65 [4]. However insurance status may be an important variable in the decision to undergo knee replacement surgery in other countries.

The addition body mass index significantly improved the predictive model for knee replacement. These findings confirm and extend the findings of de Guia et al. [11] and others [12] who reported younger patients who were overweight or obese had significantly increased risk of knee replacement. Similarly Grotle et al. [13] reported that body mass index was associated with self-reported osteoarthritis of the knee over 10-years of follow-up. However in a smaller study of participants with end-stage knee osteoarthritis Zeni et al. [14] did not find any association with body mass index and knee replacement. These negative findings may have been due to the short term follow up (2 years), cohort selection or the inclusion of both men and women in the study. This is particularly likely as a meta-analysis reported gender differences in osteoarthritis with elderly women having a higher incidence and severity of disease [27]. Thus we have demonstrated that body mass index significantly improves prediction of knee replacements over both 10-years and the inclusion of this “modifiable” variable in the model will allow the public to assess their reduction in KR risk with decreasing body weight. 

The strengths of the current study include the long-term and complete follow-up of the cohort using person-based linked hospital procedure and discharge records for participants. Western Australia is fortunate in having a system that captures complete coded diagnostic data of all public and private inpatient contacts and deaths, the Western Australian Data Linkage System (WADLS), a division of the Health Department of Western Australia. The WADLS provides a comprehensive, population-based linkage system that connects data from over 30 health-related data sets of residents of Western Australia [28]. The use of this data system allowed complete ascertainment of verified adverse events for patients in cohort studies, independently of patient report. The validity of Western Australian HMDS data has been exhaustively verified with over 250 publications [28]. This data in conjunction with anthropometrical measures, medications history and questionnaires regarding joint pain allowed detailed assessment of the role of simply evaluated patient self-reported knee joint replacement risk factors for KR over a long period of follow up. 

A limitation of the study was the lack of radiography at baseline for the diseased knee joints which has in other studies has been shown to be predictive of KR in longitudinal studies of participants with osteoarthritic joint pain [14,29,30,31]. The inclusion of radiography may have further improved the discrimination of the model. Despite this and the inherent variability in the decision to undergo surgery, simply assessed measures predicted long term knee replacement well in this high risk population. Further replication is needed to externally validate the model in other population based cohorts and countries where socioeconomic status may contribute to the model. Despite this we see the development of a predictive model as an important first step in the development of strategies to reduce the burden of joint replacement surgery. This early identification will allow targeted interventions in the high risk group similar to other chronic disease risk calculators.

In conclusion widespread use of a population-based risk calculator for early identification of 10-year KR risk may allow identification of high-risk individuals who can then seek radiological assessment and be targeted for improved early treatment options. This model may allow clinical researchers to identify high risk individuals from large population based cohorts for randomized controlled trials of novel treatments to prevent progression to knee replacement. 

## Supporting Information

Figure S1 **Receiver operator characteristic (ROC) curve for the 5-variable predictive model at 48 months compared to the weighted WOMAC score.** 5-variable model C-statistic 0.810 ± 0.023 (black line) and WOMAC 0.748 ± 0.030 (red line).
(TIFF)

Table S1 **Characteristics at 48 months.**
(DOCX)

## References

[1] Australian Institute of Health and Welfare (2008) Arthritis and Osteoporosis in Australia 2008. Canberra: AIHW.

[2] Jenkins PJ, Clement ND, Hamilton DF, Gaston P, Patton JT et al. (2013) Predicting the cost-effectiveness of total hip and knee replacement: a health economic analysis. Bone Joint J 95-B: 115-121. doi:10.1302/0301-620X.95B1.29835. PubMed: 23307684. 23307684

[3] Labek G, Thaler M, Janda W, Agreiter M, Stöckl B (2011) Revision rates after total joint replacement: cumulative results from worldwide joint register datasets. J Bone Joint Surg Br 93: 293-297. PubMed: 21357948. 21357948 10.1302/0301-620X.93B3.25467

[4] Kim S (2008) Changes in surgical loads and economic burden of hip and knee replacements in the US: 1997-2004. Arthritis Rheum 59: 481-488. doi:10.1002/art.23525. PubMed: 18383407. 18383407

[5] Dixon T, Shaw M, Ebrahim S, Dieppe P (2004) Trends in hip and knee joint replacement: socioeconomic inequalities and projections of need. Ann Rheum Dis 63: 825-830. doi:10.1136/ard.2003.012724. PubMed: 15194578. 15194578 PMC1755069

[6] Cameron AJ, Welborn TA, Zimmet PZ, Dunstan DW, Owen N et al. (2003) Overweight and obesity in Australia: the 1999-2000 Australian Diabetes, Obesity and Lifestyle Study (AusDiab). Med J Aust 178: 427-432. PubMed: 12720507. 12720507 10.5694/j.1326-5377.2004.tb05998.x

[7] Cameron AJ, Dunstan DW, Owen N, Zimmet PZ, Barr EL et al. (2009) Health and mortality consequences of abdominal obesity: evidence from the AusDiab study. Med J Aust 191: 202-208. PubMed: 19705980. 19705980 10.5694/j.1326-5377.2009.tb02753.x

[8] Chen J, Devine A, Dick IM, Dhaliwal SS, Prince RL (2003) Prevalence of lower extremity pain and its association with functionality and quality of life in elderly women in Australia. J Rheumatol (submitted). PubMed: 14719214. 14719214

[9] Manninen P, Riihimäki H, Heliövaara M, Mäkelä P (1996) Overweight, gender and knee osteoarthritis. Int J Obes Relat Metab Disord 20: 595-597. PubMed: 8782738. 8782738

[10] Felson DT, Anderson JJ, Naimark A, Walker AM, Meenan RF (1988) Obesity and knee osteoarthritis. The Framingham Study. Ann Intern Med 109: 18-24. doi:10.7326/0003-4819-109-1-18. PubMed: 3377350. 3377350

[11] de Guia N, Zhu N, Keresteci M, Shi JE (2006) Obesity and joint replacement surgery in Canada: findings from the Canadian Joint Replacement Registry (CJRR). Healthc Policy 1: 36-43. PubMed: 19305668. 19305668 PMC2585344

[12] Wang Y, Simpson JA, Wluka AE, Teichtahl AJ, English DR et al. (2009) Relationship between body adiposity measures and risk of primary knee and hip replacement for osteoarthritis: a prospective cohort study. Arthritis Res Ther 11: R31. doi:10.1186/ar2636. PubMed: 19265513. 19265513 PMC2688176

[13] Grotle M, Hagen KB, Natvig B, Dahl FA, Kvien TK (2008) Obesity and osteoarthritis in knee, hip and/or hand: an epidemiological study in the general population with 10 years follow-up. BMC Musculoskelet Disord 9: 132. doi:10.1186/1471-2474-9-132. PubMed: 18831740. 18831740 PMC2573886

[14] Zeni JA Jr., Axe MJ, Snyder-Mackler L (2010) Clinical predictors of elective total joint replacement in persons with end-stage knee osteoarthritis. BMC Musculoskelet Disord 11: 86. doi:10.1186/1471-2474-11-86. PubMed: 20459622. 20459622 PMC2877653

[15] Prince RL, Devine A, Dhaliwal SS, Dick IM (2006) Effects of calcium supplementation on clinical fracture and bone structure: results of a 5-year, double-blind, placebo-controlled trial in elderly women. Arch Intern Med 166: 869-875. doi:10.1001/archinte.166.8.869. PubMed: 16636212. 16636212

[16] Britt H, Scahill S, Miller G (1997) ICPC PLUS for community health? A feasibility study. Health Inf Manag 27: 171-175. PubMed: 10178424. 10178424 10.1177/183335839802700406

[17] Devine A, Dhaliwal SS, Dick IM, Bollerslev J, Prince RL (2004) Physical activity and calcium consumption are important determinants of lower limb bone mass in older women. J Bone Miner Res 19: 1634-1639. doi:10.1359/JBMR.040804. PubMed: 15355558. 15355558

[18] Bruce DG, Devine A, Prince RL (2002) Recreational physical activity levels in healthy older women: the importance of fear of falling. J Am Geriatr Soc 50: 84-89. doi:10.1046/j.1532-5415.2002.50012.x. PubMed: 12028251. 12028251

[19] McArdle WD, Katch FI, Katch VL (1991) Energy, nutrition and human performance. Philadelphia, PA: Lea & Febiger.

[20] Pollock ML, Wilmore JH, Fox SM (1978) Health and fitness through physical activity. New York, NY: Wiley.

[21] World Health Organization. (1977) Manual of the international statistical classification of diseases, injuries, and causes of death : based on the recommendations of the ninth revision conference, 1975, and adopted by the Twenty-ninth World Health Assembly. Geneva: World Health Organization 2v. p.

[22] World Health Organization. (2004) ICD-10 : international statistical classification of diseases and related health problems : tenth revision. Geneva: World Health Organization 3v. p.

[23] Bellamy N (2002) WOMAC user's guide V. Queensland: The University of Queensland Faculty of Health Sciences.

[24] Ensrud KE, Lui LY, Taylor BC, Schousboe JT, Donaldson MG et al. (2009) A comparison of prediction models for fractures in older women: is more better? Arch Intern Med 169: 2087-2094. doi:10.1001/archinternmed.2009.404. PubMed: 20008691. 20008691 PMC2811407

[25] Donaldson MG, Cawthon PM, Schousboe JT, Ensrud KE, Lui LY et al. (2011) Novel methods to evaluate fracture risk models. J Bone Miner Res 26: 1767-1773. doi:10.1002/jbmr.371. PubMed: 21351143. 21351143 PMC3544194

[26] Wilson PW, D'Agostino RB, Levy D, Belanger AM, Silbershatz H et al. (1998) Prediction of coronary heart disease using risk factor categories. Circulation 97: 1837-1847. doi:10.1161/01.CIR.97.18.1837. PubMed: 9603539. 9603539

[27] Srikanth VK, Fryer JL, Zhai G, Winzenberg TM, Hosmer D et al. (2005) A meta-analysis of sex differences prevalence, incidence and severity of osteoarthritis. Osteoarthritis Cartilage 13: 769-781. doi:10.1016/j.joca.2005.04.014. PubMed: 15978850. 15978850

[28] Holman CD, Bass AJ, Rosman DL, Smith MB, Semmens JB et al. (2008) A decade of data linkage in Western Australia: strategic design, applications and benefits of the WA data linkage system. Aust Health Rev 32: 766-777. doi:10.1071/AH080766. PubMed: 18980573. 18980573

[29] Gossec L, Tubach F, Baron G, Ravaud P, Logeart I et al. (2005) Predictive factors of total hip replacement due to primary osteoarthritis: a prospective 2 year study of 505 patients. Ann Rheum Dis 64: 1028-1032. doi:10.1136/ard.2004.029546. PubMed: 15640268. 15640268 PMC1755580

[30] Conaghan PG, D'Agostino MA, Le Bars M, Baron G, Schmidely N et al. (2010) Clinical and ultrasonographic predictors of joint replacement for knee osteoarthritis: results from a large, 3-year, prospective EULAR study. Ann Rheum Dis 69: 644-647. doi:10.1136/ard.2008.099564. PubMed: 19433410. 19433410

[31] Lane NE, Nevitt MC, Hochberg MC, Hung YY, Palermo L (2004) Progression of radiographic hip osteoarthritis over eight years in a community sample of elderly white women. Arthritis Rheum 50: 1477-1486. doi:10.1002/art.20213. PubMed: 15146417. 15146417

