# Supplementary figures and images for: A Predictive Model for Knee Joint Replacement in Older Women
Source: PLoS One. 2013 Dec 11;8(12):e83665. doi: 10.1371/journal.pone.0083665 (PMC3859639; doi:10.1371/journal.pone.0083665)

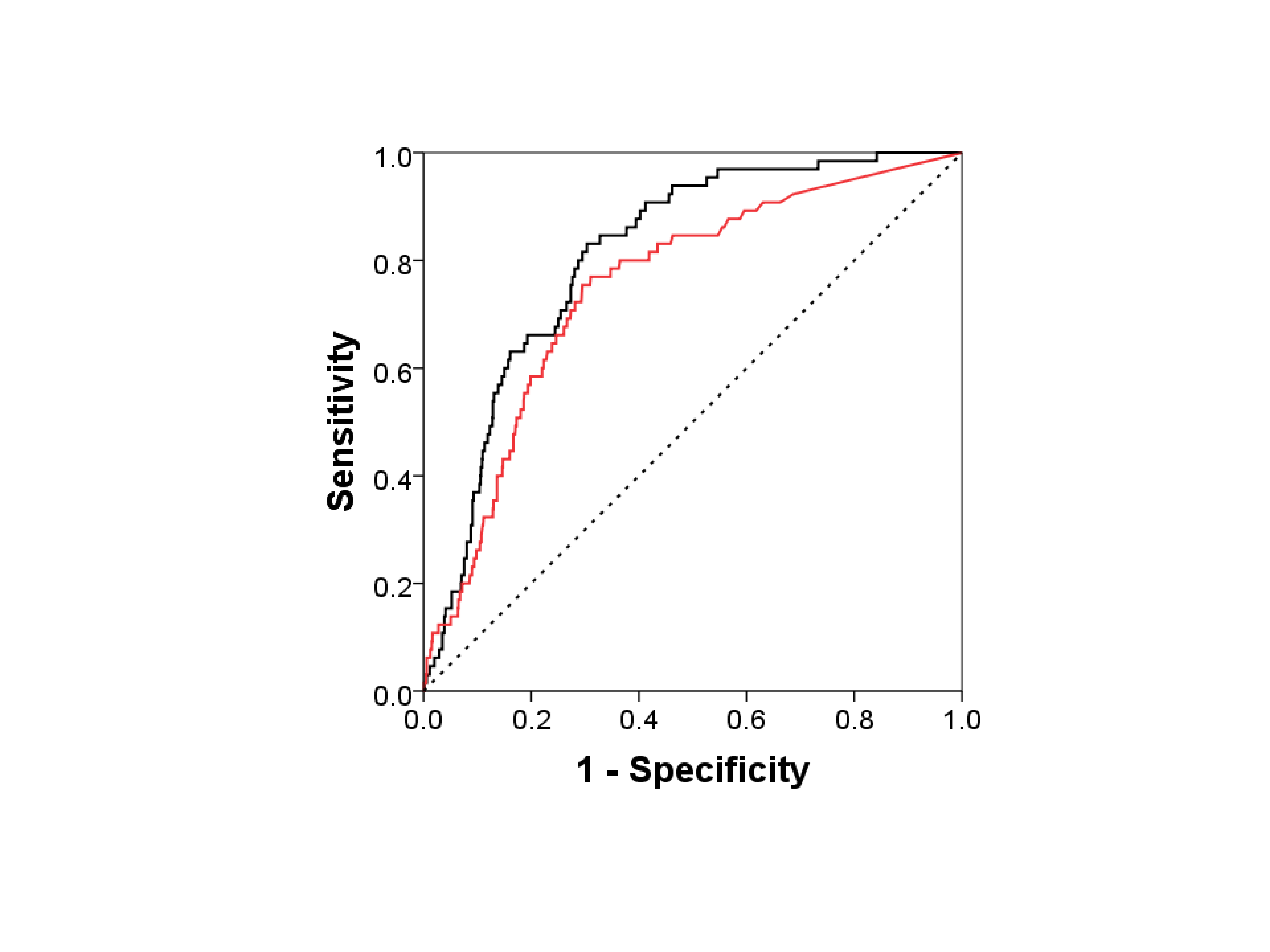

Supplement: Figure S1 — Receiver operator characteristic (ROC) curve for the 5-variable predictive model at 48 months compared to the weighted WOMAC score. 5-variable model C-statistic 0.810 ± 0.023 (black line) and WOMAC 0.748 ± 0.030 (red line). (TIFF) [file pone.0083665.s001.tiff]
