# Supplementary material for: A Predictive Model for Knee Joint Replacement in Older Women
Source: PLoS One. 2013 Dec 11;8(12):e83665. doi: 10.1371/journal.pone.0083665 (PMC3859639; doi:10.1371/journal.pone.0083665)
Supplement: Table S1 — Characteristics at 48 months. (DOCX) [file pone.0083665.s002.docx]

**Supplementary Data**

| **Supplementary Table 1.** Characteristics at 48 months | | | |
| --- | --- | --- | --- |
| **Characteristics** | **No knee replacement**  **(n = 1,054)** | **6-year knee replacement**  **(n = 65)** | **P**  **value** |
| **Age (years)** | 79.1 ± 2.6 | 79.0 ± 2.4 | 0.771 |
| **Knee pain** |  | | |
| **Infrequent** | **705 (66.9)** | **12 (18.5)** | **<0.001** |
| **Frequent** | **185 (17.6)** | **19 (29.2)** |  |
| **Daily** | **164 (15.6)** | **34 (52.3)** |  |
| **Analgesia use for joint pain (yes)** | **360 (34.4)** | **35 (53.8)** | **0.002** |
| **Previous knee replacement (yes)** | **43 (4.1)** | **9 (13.8)** | **<0.001** |
| **Body mass index (kg/m^2^)** | **27.1 ± 4.7** | **29.5 ± 4.5** | **<0.001** |
| All values are mean ± standard deviation for continuous variables or number and percentage for categorical variables. | | | |
